# Grating (Moiré) Microinterferometric Displacement/Strain Sensor with Polarization Phase Shift

**DOI:** 10.3390/s24092774

**Published:** 2024-04-26

**Authors:** Leszek Sałbut, Dariusz Łukaszewski, Aleksandra Piekarska

**Affiliations:** Warsaw University of Technology, 00-661 Warsaw, Poland; d.lukaszewski@mchtr.pw.edu.pl (D.Ł.); aleksandra.piekarska.stud@pw.edu.pl (A.P.)

**Keywords:** grating (moiré) interferometry, waveguide sensor, polarization fringe phase shift, displacement measurement

## Abstract

Grating (moiré) interferometry is one of the well-known methods for full-field in-plane displacement and strain measurement. There are many design solutions for grating interferometers, including systems with a microinterferometric waveguide head. This article proposes a modification to the conventional waveguide interferometer head, enabling the implementation of a polarization fringe phase shift for automatic fringe pattern analysis. This article presents both the theoretical considerations associated with the proposed solution and its experimental verification, along with the concept of in-plane displacement/strain sensing using the described head.

## 1. Introduction

Grating interferometry (GI), known in the relevant literature as High-Sensitivity Moiré Interferometry, is a full-field optical method used for in-plane displacement and strain measurement [1,2]. In addition to its high sensitivity, GI has a unique feature compared to other displacement measurement methods—the specimen grating retains the “memory” of its initial state, which means that all successive measurements have the same reference (the moment when the grating was attached to the specimen surface), which allows one to obtain information about the cumulative displacement between the measurement periods and enables the monitoring of the state of the tested specimen at any time. Measurement systems and sensors based on GI have many applications as they can be used in the study of composite materials [3], in electronics and MEMS micro-elements [4], in joint analysis [5], in biomechanics [6], etc. [7].

**Figure 1:**
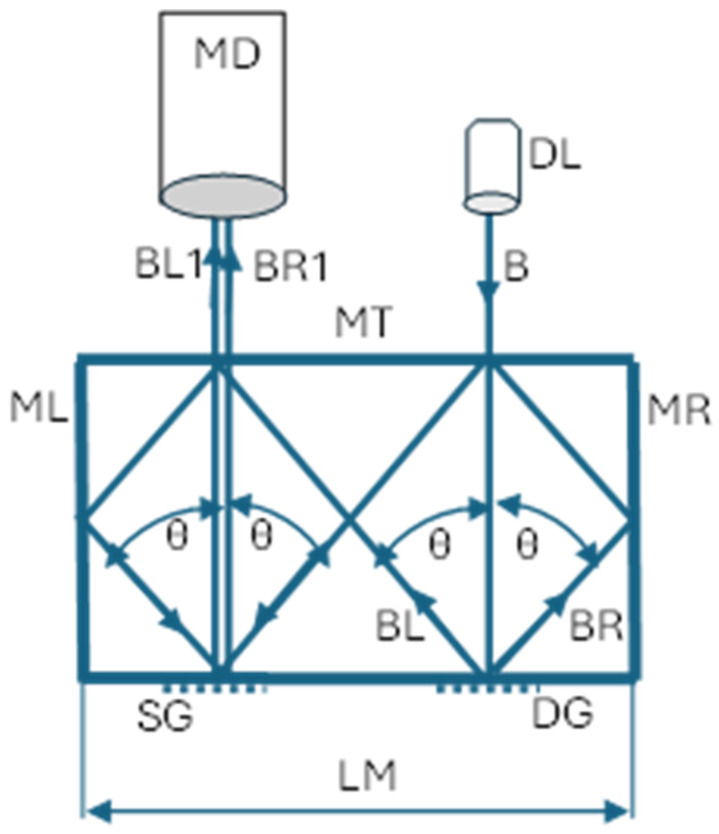
Conceptual scheme of grating microinterferometric sensor in a conventional configuration. DL—laser diode; B—collimated laser beam; ML, MT, and MR—reflective surfaces; DG—diffraction grating; SG—specimen grating; BR and BL—beams diffracted by DG grating; BL1 and BR1—beams diffracted by SG grating; θ—diffraction angle fulfilling the grating equation, which is as follows: sin θ = λ/d; and MD—matrix detector.

Grating interferometry has seen numerous design solutions over the years [8,9]. Among these solutions, sensors incorporating a microinterferometric waveguide head [10] have shown significant promise. The concept of the conventional waveguide grating microinterferometric sensor is schematically illustrated in Figure 1. The microinterferometric head comprises reflective surfaces—the top (MT), right (MR), and left (ML) surfaces—and a diffraction grating (DG). A specimen grating (SG) with the same spatial frequency as the DG is affixed to the object under examination. A collimated light beam (B) from a diode laser (DL) illuminates the diffraction grating, causing diffraction. The beam of the first positive diffraction order, after reflecting off the MR surface and then the MT surface, illuminates the specimen grating at the angle θ of the first diffraction order. The beam of the first negative diffraction order, after reflecting off the MT surface and then the ML surface, symmetrically illuminates the specimen grating. In this scenario, beams BL1 and BR1, diffracted by the specimen grating, propagate along the normal grating surface and cause interference. The intensity distribution, captured by a video camera (MD), is described by the following equation:

I(x,y) ≈ 1 + cos{4π/d u(x,y)},(1)

where d is the period of the specimen grating and u(x,y) is the function describing the in-plane displacements of the object under test.

This implies that the recorded interferogram contains information about the distribution of in-plane displacements across the entire field of view. The sensitivity of this measurement is high, equivalent to half the period of the grating (d/2). For a typical grating with a spatial frequency of *f* = 1200 lines/mm, the basic sensitivity is approximately d/2 = 1/2*f* = 0.417 µm.

Owing to the symmetry of the head configuration and the identical spatial frequencies of both gratings, the grating microinterferometer is achromatic and resistant to vibrations [11].

To analyse the obtained interferograms, various methods of automatic fringe pattern analysis (AFPA) can be employed, such as the Fourier Transform, Temporary Phase Shifting (TPS), and Carrier Frequency Phase Shifting (CFPS) [12,13]. The TPS method is often preferred in many applications. It necessitates the registration of at least three interferograms with fringes shifted by a known phase. In a displacement sensor with a conventional grating microinterferometer head, the phase shift can essentially only be achieved by a very precise movement of the head over the specimen grating. This is a complex and costly solution. Moreover, this sequential acquisition of interferograms over time requires highly stable conditions, which limits the use of the sensor to measurements of static objects.

These challenges can be addressed by using the Polarization Phase Shifting (PPS) method with a specialized video camera equipped with a polarization-sensitive subpixel mask [14]. Regrettably, the conventional grating microinterferometer head is insensitive to the polarization state due to the equal number of reflections of the propagating beams. This implies that the polarization state of the output beams will be identical for each polarization of the illuminating beam, rendering the implementation of the PPS method impossible.

The next section of this paper introduces a novel modification to the grating microinterferometry head, which enables the implementation of the polarization method to shift the phases of interference fringes.

## 2. Concept of a Grating Microinterferometric Head with Polarization Phase Shift

**Figure 2:**
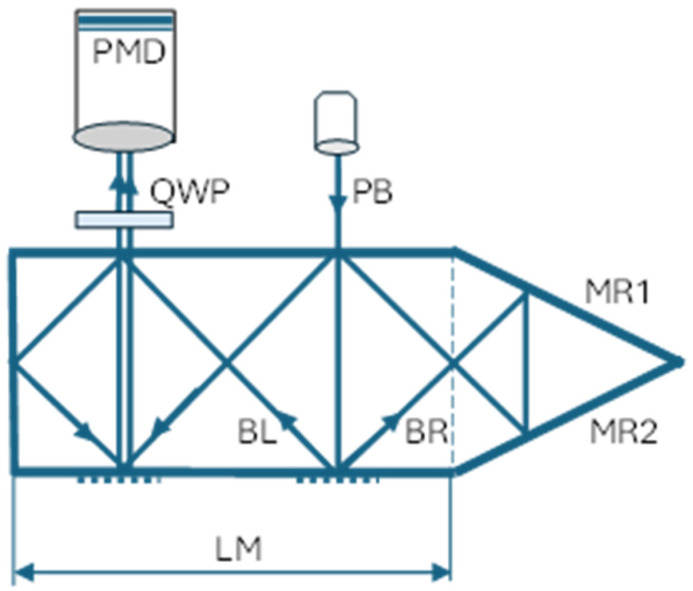
Conceptual scheme of grating microinterferometric sensor in the new configuration. DL—laser diode; PB—polarized laser beam; MR1 and MR2—reflective surfaces; BR and BL—diffracted beams; QWP—quarter-wave plate; and PMD—matrix detector with polarization mask.

The proposed new waveguide grating microinterferometer is schematically depicted in Figure 2. The configuration of this microinterferometric head is almost the same as that of the conventional head described earlier, although there is one key difference: the MR reflective surface has been replaced by two reflective surfaces (MR1 and MR2). The optical path of the BR beam is now extended, and notably, the number of specular reflections is one reflection greater than the number of reflections of the BL beam. 

A full analysis of the influence of the reflecting surface material on the changes in beam polarization after reflection is described in the next section. For now, we will ignore the influence of reflecting surfaces on the wave’s amplitude coefficients and only take into account the fact that after a light wave is reflected, the phase of the component perpendicular to the plane of incidence changes by 180 degrees. Consequently, in the event of the linear polarization of the illuminating beam at an angle of +45 degrees to the plane of incidence, the BR beam, after three reflections, will be linearly polarized at the same angle of +45 degrees. Conversely, the BL beam, after two reflections, will be linearly polarized at an angle of −45 degrees. This implies that the output beams have linear and mutually orthogonal polarizations. 

It is now feasible to employ a phase shift polarization system, which has been documented in the literature and comprises a quarter-wave plate and a rotating polarizer [15] or a matrix of polarizers with the appropriate orientation integrated with the pixels of the photosensitive matrix detector with the polarization mask (PMD) [16].

When a quarter-wave plate (QWP) is inserted at an angle of 45 degrees to the directions of the mutually orthogonal linear polarizations of the output beams, the polarization of these beams behind the quarter-wave plate becomes circular, left-handed, and right-handed, respectively. Subsequently, after passing through a polarizer oriented at an angle α, the beams will be linearly polarized in the same direction but with a phase shift of 2α. The beams can now interfere, and the observed intensity distribution can be described by the following equation:

I(x,y) ≈ 1 + cos{4π/d u(x,y) + 2α},(2)

where α is the orientation angle of the polarizer axis.

Despite the simplicity of this head modification, it introduces a significant improvement, namely, the fact that it allows for the application of the automated Temporary Phase Shifting (TPS) method for analysing the interference image captured in a single frame. This enhancement not only increases the measurement accuracy but also expands the sensor’s applicability to the study of time-varying objects. However, due to the difference in the optical paths of the propagating beams, the proposed microinterferometer loses its achromatic property, necessitating the use of a laser as the light source. This trade-off is a crucial aspect to consider in the practical implementation of the proposed system.

## 3. Design and Numerical Simulation

Building upon the concept delineated in the preceding section, an actual grating microinterferometer head was designed and developed. This head is tailored for use with reflection diffraction gratings at a spatial frequency of 1200 lines/mm and with laser light with a wavelength of λ = 632.8 nm. This means that will be possible to measure in-plane displacements with a basic sensitivity of d/2 = 0.417 µm/fringe, and after using the phase shifting method, with a theoretical resolution of 0.01 of the fringe, i.e., about 4 nm. The measurement field, limited by the input and output windows, is as follows: 2 mm × 2 mm. The spatial resolution depends on the resolution of the photosensitive matrix, and for the proposed camera, it is 2448 × 2048 pixels [16]. The framerate is equal to 24 FPS.

**Figure 3:**
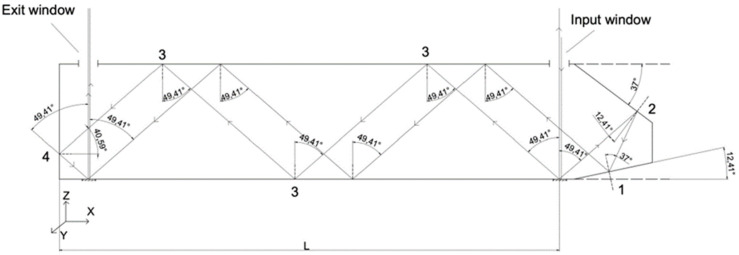
Geometry of the proposed grating microinterferometric head: The numbers 1, 2, 3, and 4 indicate the surfaces reflecting the beams at the following angles: θ_1_ = 12.41°, θ_2_ = 37°, θ_3_ = 49.41°, and θ_4_ = 40.59°, respectively.

The head is a type of cavity waveguide that has mirror surfaces coated with an aluminium layer. It is also feasible for the beams to enter and exit through windows in the upper mirror, resulting in an asymmetry at the MR1/MR2 corner. Owing to the necessity to accommodate the lighting and detection system, the length (L) of the designed head is twice the length (LM) of the basic head module depicted in Figure 2. It is worth noting that the length of the head can be extended by incorporating additional basic modules. The final geometry of the developed head is illustrated in Figure 3 and is as follows: a length of 50.8 mm and a height of 11.6 mm. 

To verify the polarization properties of the microinterferometer head designed in this manner, numerical simulations were conducted using the MATLAB v. R2023b platform. Jones matrices were employed to analyse the polarization state of both output beams [17,18]. It is important to note that this analysis sufficiently demonstrated that a polarization state of the input beam exists, for which the output beams will be linearly and orthogonally polarized relative to each other. This can be verified by analysing the contrast of the interference fringes, which, for the aforementioned situation, should diminish to zero.

Using the Jones matrix, the polarization state of the output beams can be described by the following equations:

(3) $$\left[E_{1}\right]=\left[S\right]\cdot \left[J_{3}\right]\cdot \left[J_{3}\right]\cdot \left[J_{3}\right]\cdot \left[J_{2}\right]\cdot \left[J_{1}\right]\cdot \left[S\right]\cdot [E_{0}]$$

(4) $$\left[E_{2}\right]=\left[S\right]\cdot \left[J_{4}\right]\cdot \left[J_{3}\right]\cdot \left[J_{3}\right]\cdot \left[J_{3}\right]\cdot \left[S\right]\cdot [E_{0}]$$

where

- [*E*_1_] and [*E*_2_] are the matrixes describing the amplitudes and phase of the output beams:

(5) $$\left[E_{1}\right]=\left[\begin{array}{c} E_{1x}\\ E_{1y}e^{i\delta_{1}} \end{array}\right],\;\left[E_{2}\right]=\left[\begin{array}{c} E_{2x}\\ E_{1y}e^{i\delta_{2}} \end{array}\right]$$

- [*E*_0_] is the matrix describing the amplitudes and phase of the illuminating beam:

(6) $$[E_{0}]=\left[\begin{array}{c} E_{0x}\\ E_{0y}e^{i\delta_{we}} \end{array}\right]$$

- [*S*] is the matrix describing the influence of the diffraction grating on the polarization of the diffracted beam

(7) $$\left[S\right]=\left[\begin{array}{cc} s^{+} & 0\\ 0 & s^{\|}e^{-i\delta_{s}} \end{array}\right]$$

The coefficients were determined experimentally for the reflection grating at a spatial frequency of 1200 lines/mm by measuring the intensity of the diffracted beam under illumination with a linearly polarized beam perpendicular and parallel to the grid and are as follows: *s*^+^ = 0.18 and *s*^‖^ = 0.14, respectively. The phase was estimated based on the literature [19], as follows: *δ_s_* = 1.22 rad.

- [*J_k_*] are the matrixes describing the influence of the aluminium mirrors’ reflection:

(8) $$\left[J_{k}\right]=\left[\begin{array}{cc} {r_{k}}^{\|} & 0\\ 0 & {r_{k}}^{+}e^{-i\delta_{zk}} \end{array}\right]$$

The index *k* denotes the matrix for the following incident angles: *θ*_1_ = 12.41°, *θ*_2_ = 37°, *θ*_3_ = 49.41°, and *θ*_4_ = 40.59° (see Figure 3). The values of the amplitude components and the phase for each angle of incidence were determined using Fresnel’s formulas, assuming the refractive index of aluminium is as follows: *n* = 1.37289 + *i*7.617691. We also assumed that the refractive index of the medium (air) between the reflecting surfaces is as follows: *n* = 1.0003.

Then, the intensity was determined numerically using the following relationship:

(9) $$I=Tr\left[\left(E_{1}+E_{2}\right)\;x\;{\left(E_{1}+E_{2}\right)}^{H}\right],$$

where “x” denotes the Kronecker product of the matrixes and “*H*” represents the Hermitian conjugate of the matrix.

Finally, the contrast of the interference fringes was determined based on the following standard equation:

(10) $$C=\frac{I_{max}-I_{min}}{I_{max}+I_{min}}$$

**Figure 4:**
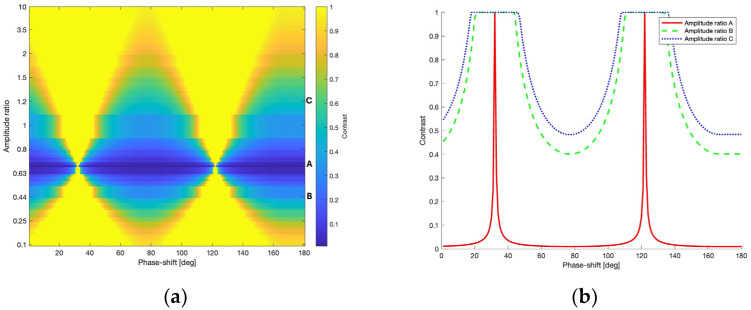
The dependence of the contrast of the interference fringes on the ratio of the amplitude components and the phase difference of the illuminating beam: (**a**) 2D diagram and (**b**) plot for amplitude ratios of 0.7 (A), 0.44 (B), and 1.2 (C).

The calculations were carried out using various ratios of the components of the illuminating beam (*E*_0*x*_/*E*_0*y*_), ranging from 0.1 to 10 for all combinations of *E*_0*x*_ and *E*_0*y*_ amplitude values, both in the range of 0.1 to 1 with a step of 0.1, and with a phase difference *δ_we_* in the range of 0 to 180°. The result is presented in Figure 4a in the form of a 2D diagram.

Then, the algorithm finds the value of the ratio of the amplitudes of the components of the illuminating beam for which the contrast reaches a minimum value close to zero. In our case, this ratio is 0.7. Figure 4b shows the contrast plot for this value (line A) and, for example, for amplitude ratios of 0.44 (line B) and 1.2 (line C). From plot (A), it can be seen that a contrast close to zero, indicating orthogonal polarizations of the output beams, can be obtained for various phase shifts between the components of the illuminating beam. The next step is to determine, based on Formulas (3) and (4), the phase shift for which the output beams are additionally linearly polarized. 

To conclude, the analysis of the acquired data revealed that the fringe contrast reaches the minimum ratio of the polarization components of the illuminating beam at 0.7 (*E*_0*X*_ = 0.7 and *E*_0*Y*_ = 1) and a phase shift of 167°. Subsequently, the polarization parameters of the output beams are as follows: the amplitude ratio for the first beam is *E*_1*X*_/*E*_1*Y*_ = 1.005, with a phase shift of *δ*_1_ = 181.30°, and for the second beam, it is *E*_2*X*_/*E*_2*Y*_ = 1.002, with *δ*_2_ = 1.52°. This implies that the beams are practically linearly polarized at angles of ±45°, rendering them mutually orthogonal, a prerequisite for implementing the PPS method. In this scenario, to ensure the appropriate polarization of the input beam, a polarization compensator (phase plate) would be necessary. However, in practice, using a half-wave plate that rotates the polarization plane of the illuminating beam by an angle of approximately arctan(0.7) ≈ 35° may be sufficient.

## 4. Experimental Work

**Figure 5:**
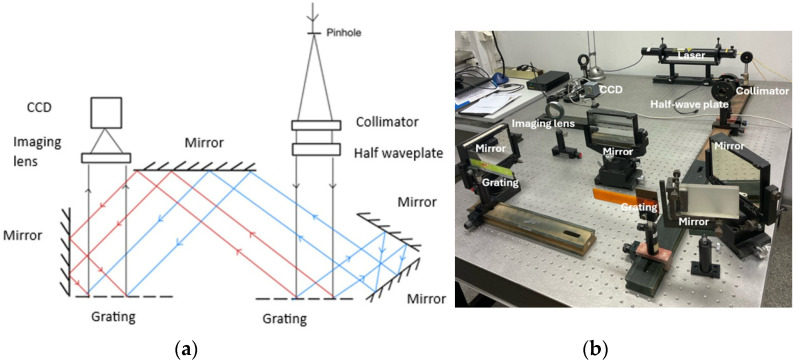
Scheme (**a**) and photograph (**b**) of the laboratory model of the grating interferometer.

**Figure 6:**
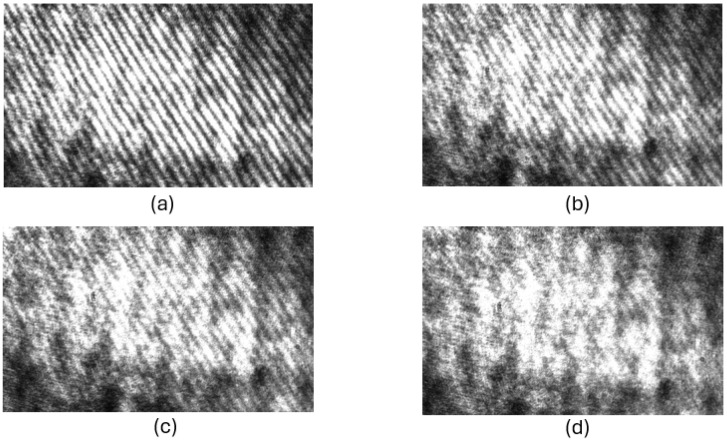
Interferograms recorded for different angles of rotation of the polarization plane of the illuminating beam: (**a**) α = 0°, (**b**) α = 10°, (**c**) α = 20°, and (**d**) α = 35°. The contrast of the interference fringes is as follows: C_1_ ≈ 0.8, C_2_ ≈ 0.5, C_3_ ≈ 0.3, and C_4_ < 0.2.

To experimentally validate the described grating microinterferometer head as a displacement sensor, preliminary experimental tests were conducted. A schematic and macro-scale photograph of the laboratory system are presented in Figure 5. Initially, the system was calibrated to achieve maximum fringe contrast. Previous analyses indicate that this condition is met when the polarization plane of the illuminating beam is either parallel or perpendicular to the plane of incidence. The interferogram registered for this case is shown in Figure 6a, and the contrast of the fringes (*C*_1_) determined based on Equation (10) is approximately 0.8.

Subsequently, a half-wave plate was utilized to rotate the polarization plane until the fringe contrast vanished. This was achieved for a half-wave plate rotation angle of 17° ± 1°, which means that the rotation of the polarization plane is approximately 35°, which is consistent with the analysis presented in the previous section. For this case, the interferogram is shown in Figure 6d, and the contrast (*C*_4_) of the fringes was determined to be less than 0.2. Interferograms for intermediate values of the rotation angle of the polarization plane of 10° and 20° are shown in Figure 6b and Figure 6c, respectively, along with the determined contrast values.

The sequence of recorded interferograms, as depicted in Figure 6, provided empirical evidence for the theoretical and numerical analyses conducted. The observed disappearance of the fringe pattern contrast, specifically in Figure 6d, validates the accuracy of the analyses and affirms the efficacy of the proposed modification to the interferometric displacement sensor head. This experimental confirmation underscores the potential of the proposed design in enhancing the performance of grating microinterferometric systems.

## 5. Conclusions

This paper presents a novel modification to the conventional waveguide grating microinterferometer head, enabling the implementation of a polarization fringe phase shift. This enhancement facilitates automatic fringe pattern analysis, thereby increasing the efficiency and accuracy of measurements. The proposed solution has undergone experimental verification, confirming its practical applicability. This work contributes to the ongoing advancements in grating interferometry by bridging the gap between theory and practice. Additionally, the simultaneous use of smart pixel sensors could further enhance the capabilities of the proposed grating microinterferometer system. These sensors, with their ability to process information at the pixel level, could potentially improve the speed and accuracy of fringe pattern analysis. This could lead to more precise displacement and strain measurements, particularly in dynamic, real-time applications. This integration represents an exciting avenue for future research and development in the field of grating interferometry. It underscores the potential of this technology to revolutionize various scientific and industrial applications. Further exploration and experimentation in this direction are needed to fully realize the benefits of this approach. 

## Data Availability

Data available upon request.
